# Quality of life and adjustment in men with prostate cancer: Interplay of stress, threat and resilience

**DOI:** 10.1371/journal.pone.0239469

**Published:** 2020-09-17

**Authors:** AnnMarie Groarke, Ruth Curtis, Jean Skelton, Jenny M. Groarke

**Affiliations:** 1 School of Psychology, National University of Ireland Galway, Galway, Ireland; 2 Centre for Improving Health-Related Quality of Life, School of Psychology, Queen’s University Belfast, Belfast, United Kingdom; Iranian Institute for Health Sciences Research, ISLAMIC REPUBLIC OF IRAN

## Abstract

**Purpose:**

Diagnosis and treatment of prostate cancer can generate many challenges which impact on adjustment, so understanding the psychosocial factors which contribute to individual vulnerability to poor adaptation warrants further investigation. This study investigates stress and masculine identity threat as predictors of quality of life and emotional adjustment in men with localized prostate cancer and the role of resilience as a potential protective psychological factor.

**Methods:**

Participants were invited to complete a survey study via online prostate cancer forums. Participants were 204 men ranging in age from 44–88 years (M = 65.24±7.51) and who were diagnosed with early localized prostate cancer within the previous five years. Measures used included the Perceived Stress Scale, Cancer-Related Masculine Threat Scale and the Conor-Davidson Resilience Scale. Using a cross-sectional online survey design, the extent to which perceived stress, masculine threat and psychological resilience are associated with quality of life, positive and negative affect and distress was assessed.

**Results:**

Hierarchical regression analysis demonstrated that perceived stress accounted for 26%-44% of variance on quality of life and adjustment indices, with high stress associated with low mood and poor quality of life. Low masculine threat and high resilience predicted better quality of life and emotional adjustment accounting for between 1–7% of the variance. Resilience moderated the relationship between stress and distress and mediated the association between masculine threat and distress and negative affect.

**Conclusion:**

Perceived stress was the most powerful predictor in the model and findings suggest it contributes significantly to functional and affective status in survivors of prostate cancer. Psychological resilience is a protective factor which buffers the negative effect of stress and masculine identity threat on emotional adjustment. Findings indicate that men should be screened as part of the diagnostic and treatment process for high perceived stress and low resilience to identify those at risk for poor adjustment during survivorship.

## Introduction

Incidence of prostate cancer worldwide is increasing as screening becomes more available and life expectancy rises [1]. The majority of men, however, are diagnosed with localised disease [2], and the types of treatments available include radical prostatectomy (RP) external beam radiation (EBR), brachytherapy, hormone therapy, a combination of these, or active surveillance. Treatment-related side effects can be distressing as they include urinary, bowel and sexual dysfunction [3, 4]. Given increased survival rates and the fact that many men experience long-term side effects [5] it is essential to understand the psychosocial factors which contribute to poor emotional adjustment and quality of life in survivors.

### Quality of life in prostate cancer survivors

Several studies demonstrate a negative impact on quality of life following diagnosis and treatment of prostate cancer [3, 6–9], as well as, longer term decrements in quality of life outcomes up to 15 years later [10–13]. Better understanding of the factors that influence quality of life in the short and longer term is thus critical [8]. There is also a general consensus that there are significant differences in quality of life depending on stage of cancer and treatment [1, 3, 8]. Korfarge et al. [14] found that though many experienced physical and sexual dysfunction, they did not consider these issues when reporting their general health-related quality of life. These researchers suggest that side effects are viewed as unavoidable consequences of treatments that men must learn to accept, or that being diagnosed and treated for a potentially life-threatening disease may have resulted in a change in men’s definition of health [15]. Therefore, it is important to assess quality of life from a prostate specific perspective.

### Adjustment in prostate cancer survivors

The international Psycho-Oncology Society’s standard of quality cancer care recommends screening for emotional distress as part of routine care [16]. This along with the validation of the Distress Thermometer by Chambers [17] places emphasis on emotional outcomes in contrast to the previous research focus on functional issues. There are conflicting findings on the existence of elevated psychological distress among patients with prostate cancer. For example, one review found no difference in depression between patients and non-patients [18] and other reviews found higher levels of depression and anxiety related to prostate cancer treatment and to specific symptoms, especially pain, fatigue, bowel and sexual dysfunction [19–21]. Despite these differences, significant numbers of men do experience psychological distress [22], and thus identifying factors that ameliorate such distress is of value.

### Predictors of quality of life and adjustment

The potential trajectory of disease from diagnosis through treatment decisions to living with the consequences is, over time, inherently stressful. Individual perceptions of stress as uncontrollable and overloading may predict variability in psychological and physical adjustment even more so than the objective disease stressors [23]. Kreitler et al. [24] reported that perceived stress mediated the impact of cancer stage and treatment on quality of life in a group with diverse cancers, while higher levels of perceived stress predicted poor adjustment at diagnosis of prostate cancer [24] and at different time points up to 2 years [25–28]. This study extends that timeframe to examine the impact of stress appraisal on quality of life and adjustment up to 5 years post diagnosis.

Psychological resilience has been shown to build an individual's capacity to cope with stressors [29]. While resilience in adult cancer care has not been widely investigated, existing evidence confirms a link with psychological well-being in mixed cancer groups [30]. Recent studies with prostate cancer patients indicated that high psychological resilience was significantly and inversely correlated with depression from six months to five years post-diagnosis [31, 32] suggesting that it has a buffering effect on mood. In addition, resilience mediated the relationship between a treatment side effect (urinary incontinence) and depression [33]. Furthermore, there was a differential impact of resilience on level of depression with the relationship holding only for those with low to moderate levels of physiological chronic stress but surprisingly not for those with high levels [34]. Their study used salivary cortisol as a marker of stress, the current study extends the focus to a psychological measure of stress appraisal to clarify the moderating impact of resilience on adjustment.

Symptoms of erectile dysfunction, incontinence, and fatigue may challenge the fundamentals of traditional masculine identity [35] and this gender role threat is related to poor physical and psychological outcomes in men with cancer [36, 37]. Much of the work on masculine identity to date has focused on qualitative analysis of men’s experiences following their treatment. The development of a 25-item multidimensional measure of cancer-related masculine threat [38] now allows a quantitative assessment of identity threat alongside assessment of psychosocial adjustment. Using this measure, it was shown that the extent to which men, diagnosed within the previous two years, believe that cancer is inconsistent with their masculinity exacerbates decline in functional aspects of adjustment following cancer treatment [38]. Masculine identity threat (using a single item measure) and low resilience were also associated with higher emotional distress in newly diagnosed prostate cancer patients [39]. The present study further explicates the role of resilience in masculine threat and distress in the survivorship phase.

### Aims

The aim of the present study is (i) to examine the role and relative impact of perceived stress, cancer-related masculine threat, and psychological resilience in explaining variability on quality of life and emotional adjustment (i.e., distress, positive affect and negative affect), and (ii) to explore if psychological resilience moderates the relationship between perceived stress and adjustment (distress, negative affect) and mediates the relationship between masculine threat and adjustment (distress, negative affect). Based on theory and existing empirical evidence it is predicted that perceived stress and masculine threat will predict poorer quality of life and adjustment, resilience will predict higher quality of life and adjustment, and that resilience will buffer the relationship between perceived stress and distress.

## Method

### Procedure

The study protocol was approved by the Research Ethics Committee of the National University of Ireland, Galway and was conducted in accordance with the principles of the Declaration of Helsinki. Participants were self-selected and consisted of men who had been diagnosed with prostate cancer in the last 5 years. Participants were invited to complete a battery of web-based questionnaires via online prostate cancer forums (Cancer Support Network, The New Prostate Cancer InfoLink). Clinical Gatekeepers were sent a brief description of the study with inclusion criteria (i.e., diagnosed with localised prostate cancer within the past 5 years, no other medical diagnoses). They disseminated the information and survey link to forum users. Prospective participants read a Participant Information sheet and if they were interested in completing the online survey they provided written informed consent. The enrolment period was January 2018 to September 2018.

### Participants

Participants were 204 men who had a diagnosis of prostate cancer in the last 5 years. Exclusion criteria were prior cancer diagnosis or other co-morbidities.

### Power

A power analysis was conducted using GPower 3.1. It was estimated that 146 participants were sufficient to detect a small effect in the context of power of .95 and two-tailed alpha level of .05.

### Materials

#### Perceived Stress Scale-14 (PSS) [40]

Perceived stress was measured using the PSS. The scale consists of 14 questions that gauge stress levels over the last month using questions such as “In the last month, how often have you felt that you were unable to control the important things in your life?”. The measure is rated on a Likert scale from 0 (Never) to 4 (Very Often). Scores range from 0–56, with higher scores indicating higher perceived stress.

#### Cancer-Related Masculine Threat (CRMT) [38]

The 25-item CRMT scale measures masculine identity threat resulting from cancer by ascertaining agreement/disagreement with a series of statements, e.g., “Having cancer makes me feel like less of a man” and “Cancer is taking away my masculinity”. Responses are scored from 1 (strongly disagree) to 5 (strongly agree) and responses are then averaged, with higher scores indicating higher identity threat.

#### The Connor-Davidson Resilience Scale-10 (RISC-10) [41]

The RISC-10 was used as a measure of resilience and coping ability. Participants were asked to rate their responses to 10 items based on how they have handled times of adversity in the past month using a Likert scale ranging from 0 (not true at all) to 4 (true nearly all the time). Items include “I am able to adapt when changes occur” and “I can deal with whatever comes my way”. The score range for the scale is 0–40.

#### Positive and Negative Affect Schedule (PANAS) [42]

The PANAS consists of 20 adjectives: 10 describe positive emotions and 10 describe negative emotions. Participants indicate the extent to which they have experienced these emotions in the previous week, using a Likert scale ranging from 1 (very slightly or not at all) to 5 (extremely). Two sub-scale scores are derived, with higher scores indicating greater positive and negative affect, respectively.

#### Patient-Oriented Prostate Scale-Psychometric (PORPUS-P) [43]

Quality of life was assessed using the psychometric version of the PORPUS. This measure contains 10 questions examining the areas of pain, energy, social support, doctor communication, emotional state, urinary frequency, urinary leakage, sexual function, sexual dysfunction, and bowel problems. Questions in each section are rated on a Likert scale. In the case of pain, for example, responses range from “no pain and no disturbing body sensations” to “severe pain or disturbing body sensations that limit many activities”. Depending on the area being assessed, the Likert scale ranges from 1–4, 1–5, or 1–6. Responses are weighted and a total PORPUS score is calculated ranging from 0 to 100. Higher scores are indicative of better health-related quality of life.

#### Distress Thermometer (DT) [44]

Participant distress was measured using the DT. The DT is a widely used screening measure that assesses psychological distress at a single time point. Participants were asked to rate their current psychological distress on an 11-point Likert scale ranging from 0 (no distress) to 10 (extremely distressed).

### Data analysis

The study employed a cross-sectional design and used quantitative survey data. The study investigated the impact of psychological variables on post-cancer adjustment. The dependent variables were quality of life, positive affect, negative affect, and distress. The independent variables were perceived stress, cancer-related masculine identity threat, and resilience. The data were analysed using SPSS 24. As <5% of the data were missing, and were found to be missing completely at random (Little’s MCAR test: χ^2^_(1915)_ = 2154.59, p = 0.06) pairwise deletion was implemented [45, 46]. Descriptive information was calculated and Pearson correlation coefficients assessed relationships between predictor and outcome variables. Hierarchical regression identified the specific variables that contributed to quality of life and emotional adjustment, and subsequent mediation and moderation analyses were carried out to further probe the relationships between variables.

## Results

### Sample characteristics

As shown in Table 1, participants ranged in age from 44–88 years. The mean age of the sample was 65.24 (*SD* = 7.51) and the majority had completed third level education (82%). As the questionnaire battery was accessible online, responses were recorded from a number of different countries, but the majority of respondents were from North America (85%) and Europe (9%). Diagnosis was between 3 and 5 years ago for more than half the participants (55.4%). Participants who received treatment (86%) were categorised as having either surgery (radical prostatectomy) or adjuvant therapy (radiotherapy, brachytherapy, or hormone therapy), or both surgery and adjuvant treatment. The remainder (14%) were under active surveillance.

**Table 1:** Descriptive Statistics for all study variables.

**Predictors**	**Sample Range**	**Test Range**	**M**	**SD**	**α**
Age	44–88		65.24	7.51	
Perceived Stress	5–42	0–56	19.81	7.60	.85
Masculine Threat	1.04–4.36	1–5	2.50	0.51	.85
Resilience	11–40	0–40	29.75	5.89	.89
**Outcomes**	**Sample Range**	**Test Range**	**M**	**SD**	**α**
Quality of Life	25.00–97.50	0–100	68.68	15.00	.72
Positive Affect	13–49	0–50	31.26	7.25	.89
Distress	0–9	0–10	3.44	2.69	
Negative Affect	10–39	0–50	19.01	7.36	.91
**Time since Diagnosis (% of sample)**		**Treatment (% of sample)**			
0–12 months	16.7%	Adjuvant only		32.8%	
1–2 years	27.9%	Surgery only		31.4%	
3–5 years	55.4%	Surgery plus adjuvant treatment		22.1%	

α = Cronbach’s alpha reliability coefficient (i.e., internal consistency of measure in the current sample).

In addition to providing demographic and treatment data, participants were asked to list any major stressors they had experienced in the past year. A third of respondents (33%) reported experiencing stressful events. Multiple respondents reported several stressors. Stressors were categorised into themes and the number of times each theme was mentioned is reported: (i) Heath Stressors (41%)—chronic pain, bladder problems, residual symptoms from treatment, depression. (ii) Intra and Interpersonal Stressors (37%)—death of family members/friends, ill health of family members/friends, employment issues, life challenges of family members/friends. (iii) Relationship Stressors (9%)—marriage difficulties, separation/divorce, lack of sexual intimacy following prostate cancer treatment. (iv) Work related stressors (8%)—loss of job, high pressure of job, reduced work hours following illness. (iv) Unstable living situation (5%) which related to not having a stable home environment.

Descriptive statistics for variables included in the analysis are presented in Table 1.

Correlations between predictor and outcome variables are presented in Table 2. Perceived stress and masculine identity threat were negatively correlated with quality of life and positive affect, and were positively correlated with distress and negative affect. Resilience was positively associated with quality of life and positive affect, and negatively correlated with distress and negative affect. Surgery was associated with higher quality of life and positive affect, whereas adjuvant treatment only and surgery plus adjuvant treatment were associated with lower quality of life and higher negative affect.

**Table 2:** Correlations between predictors and outcome variables included in hierarchical regression analyses.

	Quality of Life	Positive Affect	Distress	Negative Affect
Age	0.09	0.07	-0.12	-0.25**
Time since diagnosis	0.05	0.06	0.01	-0.08
***Type of Treatment***				
Adjuvant	0.02	-0.10	-0.05	-0.16*
Surgery	0.20*	0.18*	-0.06	0.01
Surgery & Adjuvant	-0.23**	-0.09	0.13	0.18*
***Psychological variables***				
Perceived Stress	-0.58***	-0.52***	0.66***	0.70***
Masculine Threat	-0.33***	-0.39***	0.32***	0.43***
Resilience	0.49***	0.51***	-0.48***	-0.52***

*p< 0.05

**p< 0.01

***p< 0.001.

A series of 4 hierarchical multiple regression analyses were performed to examine the impact of perceived stress, masculine threat and resilience on: (1) quality of life, and (2) positive affect, (3) distress, and (4) negative affect (i.e., emotional adjustment). Demographic and medical variables were entered in the first 3 steps. Psychological variables were entered in steps 4 to 6 to examine whether they explain any additional variance in the prediction of quality of life and emotional adjustment. The results of these hierarchical multiple regressions are presented in Table 3. Table 3 presents the *F* change and Adjusted *R*^2^ change values for each step, and the standardized beta coefficients for each individual predictor.

**Table 3:** Four hierarchical regression analyses explaining quality of life and emotional adjustment by demographic, medical and psychological variables.

*Outcomes*	1. Quality of Life			2. Positive Affect			3. Distress			4. Negative Affect		
***Predictors***	β	*F*	*Adj R*^*2*^	β	*F*	*Adj R*^*2*^	β	*F*	*Adj R*^*2*^	β	*F*	*Adj R*^*2*^
**Step 1**		1.27	0.00		0.81	-0.00		2.56	0.01		10.90**	0.06
Age	-0.04			-0.01			0.00			-0.25**		
**Step 2**		0.09	0.00		0.30	0.00		0.34	0.00		0.02	0.00
Time since diagnosis	0.05			0.05			0.03			-0.02		
**Step 3**		5.73**	0.04		3.05	0.02		1.04	0.00		1.93	0.02
*Treatment*												
Adjuvant	-0.01			-0.13			-0.02			-0.11		
Surgery	0.14*			0.21**			-0.03			0.05		
Surgery & Adjuvant	-0.17*			-0.01			0.09			0.16**		
**Step 4**		86.80***	0.32		59.92***	0.26		119.17***	0.41		151.35***	0.44
Perceived Stress	-0.39***			-0.26***			0.55***			0.53***		
**Step 5**		8.11**	0.03		17.15***	0.07		4.57*	0.01		15.97***	0.04
Masculine Threat	-0.15*			-0.24***			0.13*			0.20***		
**Step 6**		77.67**	0.03		12.07**	0.04		1.85	0.00		4.04*	0.01
Resilience	0.22**			0.28**			-0.11			-0.14*		

*p< 0.05

**p< 0.01

***p< 0.001; *F* = *F* change; Adj *R*^2^ = Adjusted *R*^2^ change.

Relative to psychological variables, demographic and medical variables had little to no impact on quality of life or emotional adjustment. Overall, demographic, medical and psychological variables together predicted 42% of the variance in quality of life (*F*_(7,171)_ = 18.78, *p* < .001, Adjusted *R*^*2*^ = .42), 38% of the variance in positive affect (*F*_(7,169)_ *=* 16.03, *p* < .001, Adjusted *R*^2^ = .38), 43% of the variance in distress (*F*_(7,171)_ = 19.65, *p* < .001 Adjusted *R*^2^ = .43), and 56% of the variance in negative affect (*F*_(7,168)_ = 31.47, *p* < .001, Adjusted *R*^2^ = .56). Older age predicted less negative affect (β = -0.25) explaining 6% of the variance. Type of treatment accounted for 4% of the variance in quality of life. Having surgery predicted higher quality of life (β = 0.14), whereas, surgery plus adjuvant treatment predicted lower quality of life (β = -0.17). Perceived stress was the strongest predictor, explaining 32% of the variance in quality of life, 26% of the variance in positive affect, 41% of the variance in distress, and 44% of the variance in negative affect. Perceived stress significantly predicted lower quality of life (b = -.39), lower positive affect (β = -.26), higher distress (β = .55), and higher negative affect (β = .53). Higher levels of masculine threat predicted lower quality of life (β = -.15) and positive affect (β = -.24), but only explained a small amount of variance (3% and 7% respectively). Higher masculine threat predicted higher distress (β = .13) and negative affect (b = .20), explaining only 1 and 4% of the variance. Resilience predicted higher quality of life (β = .22), higher positive affect (β = .28), and lower negative affect (β = -.14), again explaining only a small amount of the variance (1–4%).

### Moderation analysis

In the hierarchical regression analysis, resilience was a direct predictor of quality of life, positive affect, and negative affect but accounted only for a very small amount of variance. Therefore, resilience was investigated as a moderator of the relationship between perceived stress and distress and negative affect. Hierarchical regression analysis was performed using the SPSS macro PROCESS v3.4.1 [47]. The moderating effect of the interaction between resilience and stress on negative affect was not significant (β = -.00, *t*_(185)_ = -1.31, *p* = .191).

**Fig 1:**
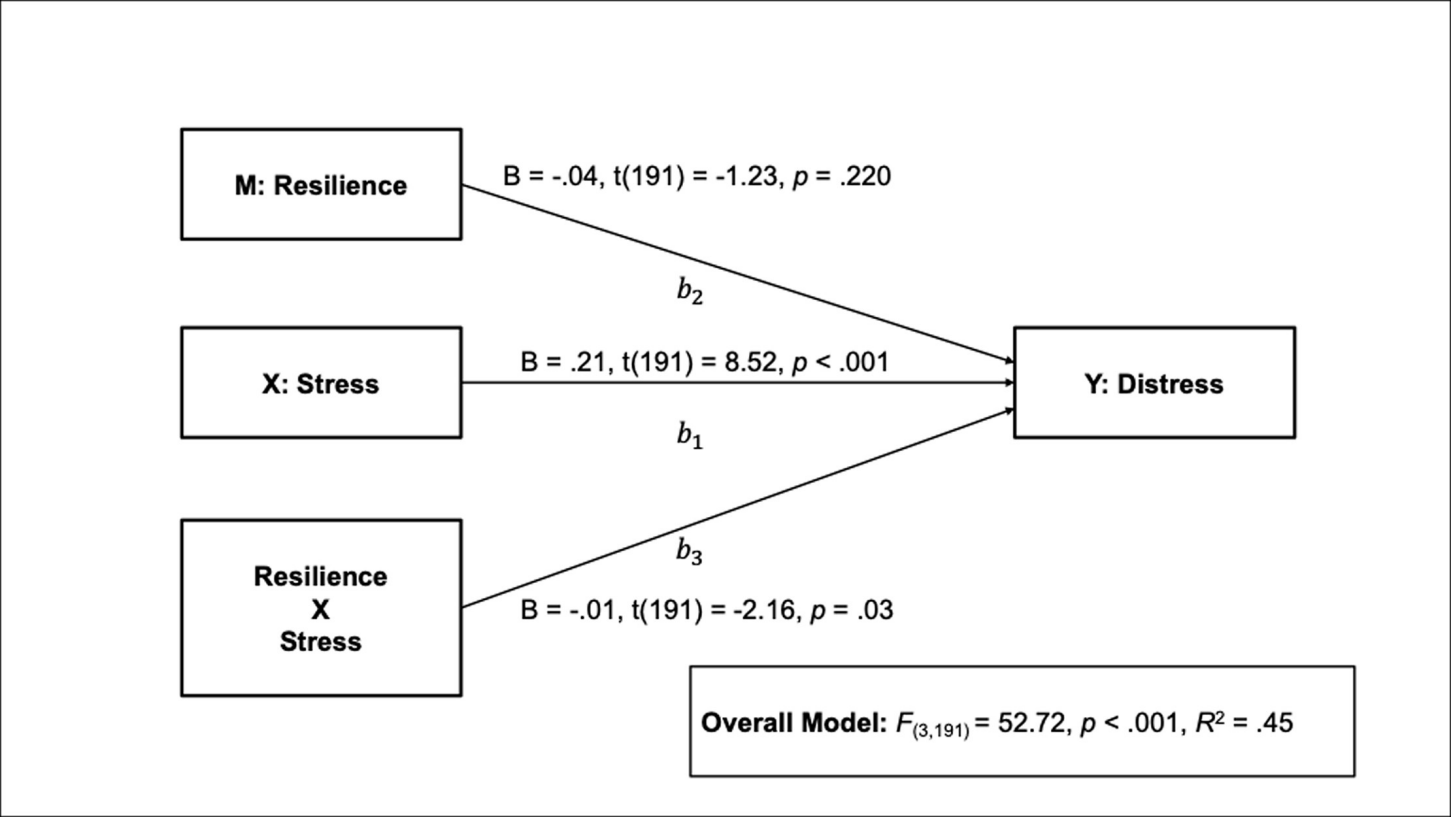
The effect of stress on distress moderated by resilience. B = unstandardized Beta coefficients.

The overall model examining the effect of perceived stress on distress moderated by resilience was significant, with 45% of the variance in distress explained by stress, resilience and their interaction (*F*_(3,191)_ = 52.72, *p* < .001, *r*^2^ = .45; see Fig 1).

**Fig 2:**
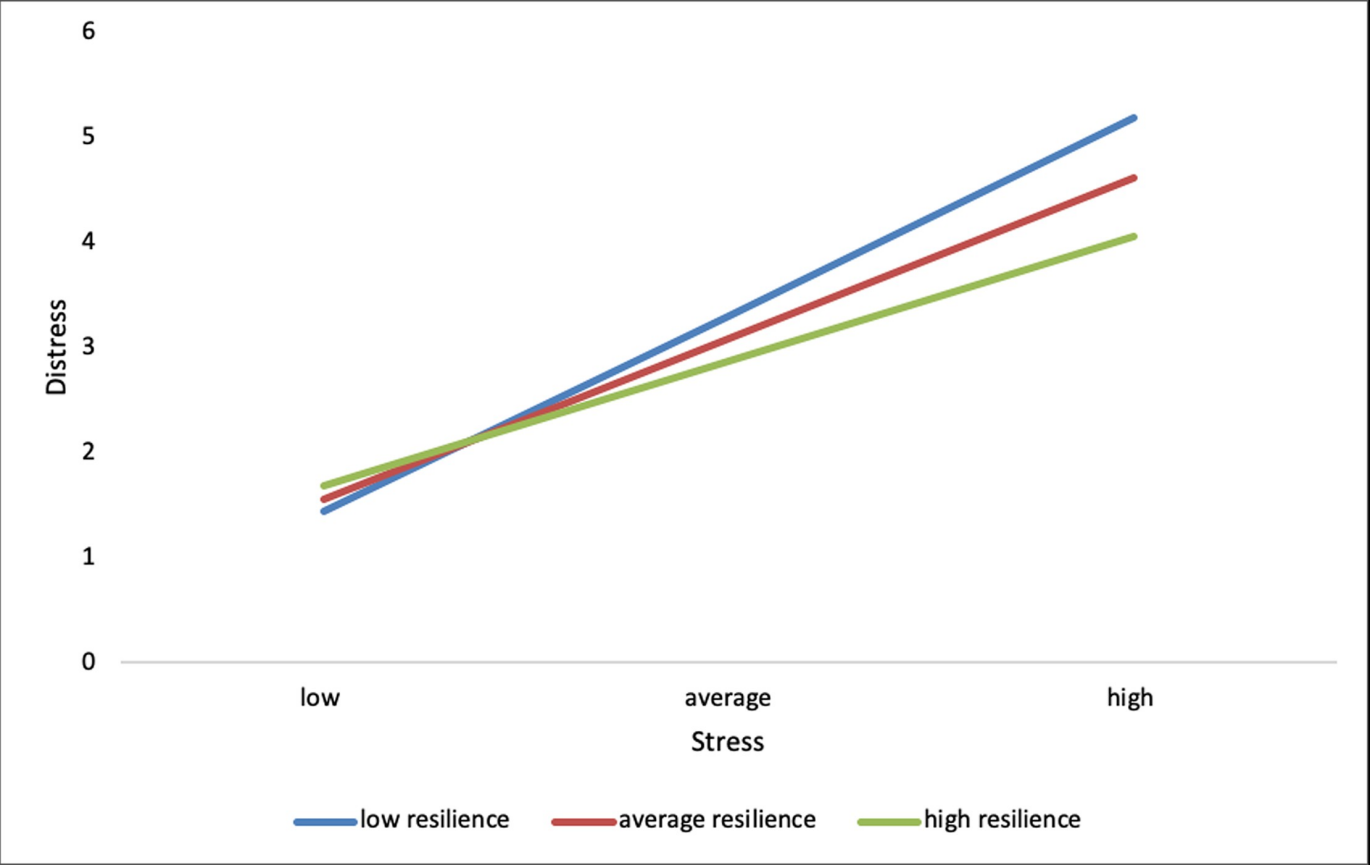
Interaction plot showing impact of increasing perceived stress on distress moderated by resilience.

The addition of the interaction was a significant change to the model, (*F*_(1,191)_ = 4.65, *p* = .032, *r*^2^ change = .013), corresponding to 1% of the overall variance explained. As perceived stress increases so does distress (path b1). However, the effects of stress on distress differed by level of resilience (path b3). For those low in resilience every one-unit increase in perceived stress increases distress scores by .24 (B = .24, *t*_(191)_ = 8.13, *p* < .001). When resilience levels are average every one-unit increase in perceived stress increases distress by .20 (B = .20, *t*_(191)_ = 8.45, *p* < .001). For high resilience levels every one-unit increase in perceived stress increases distress by .17 (B = .17, *t*_(191)_ = 5.69, *p* < .001). Examination of the interaction plot (Fig 2) shows a positive effect of increasing perceived stress on distress, such that, the impact of stress on distress is strongest when resilience is low and weakest when resilience is high.

### Mediation analysis

Cancer-related masculine threat (CRMT) predicted higher distress and negative affect, accounting for between 1 and 4% of the variance. To examine the possibility that the relationship between masculine threat and poor emotional adjustment is mediated by personal resources (resilience) a series of hierarchical regression analyses were performed using the SPSS macro PROCESS v3.4.1 [45]. Results are presented below.

It was proposed that CRMT (X) indirectly affects distress (Y) through the mediating influence of resilience (M). In step 1 of the mediation model, the regression of CRMT on distress was significant (path c: b = .31, *t*_(194)_ = 4.52, *p* < .001). Step 2 shows that the regression of CRMT (X) on the mediator, resilience, was also significant (path a: b = -.32, *t*_(194)_ = -4.77, *p* < .001). Step 3 shows that the mediator (resilience) significantly predicts distress, controlling for CRMT (path b: b = -.45, *t*_(193)_ = -7.05, *p* < .001). Step 4 of the analyses revealed that, controlling for the mediator (resilience), CRMT was still a significant but substantially weakened predictor of distress (path c’: b = .16, *t*_(193)_ = 2.50, *p* = .01).

**Fig 3:**
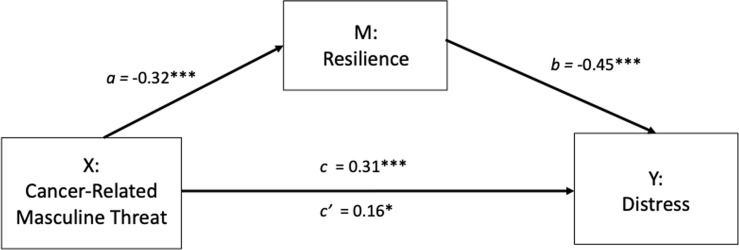
Mediation model showing the effect of masculine threat on distress is mediated by resilience. *p< 0.05, **p< 0.01, ***p< 0.001.

There was a significant indirect effect of masculine identity threat on distress through resilience (ab_cs_ = .15, BCa CI [.07, .23], see Fig 3). The Sobel test confirmed there was significant mediation (Z = 3.60, *p* < .001). The mediator could account for roughly 46% of the total effect (*P*_m_ = .46).

It was proposed that CRMT (X) indirectly affects negative affect (Y) through the mediating influence of resilience (M). In step 1 of the mediation model, the regression of CRMT on negative affect was significant (path c: b = .42, *t*_(190)_ = 6.31, *p* < .001). Step 2 shows that the regression of CRMT (X) on the mediator, resilience, was also significant (path a: b = -.31 *t*_(190)_ = -4.58, p < .001). Step 3 shows that the mediator (resilience) significantly predicts negative affect, controlling for CRMT (path b: b = -.48, *t*_(189)_ = -7.94, *p* < .001). Step 4 of the analyses revealed that, controlling for the mediator (resilience), CRMT was still a significant, albeit weaker, predictor of negative affect (path c’: b = .27, *t*_(189)_ = 4.39, *p* < .001).

**Fig 4:**
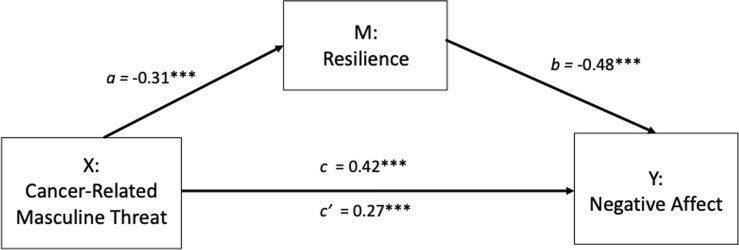
Mediation model showing the effect of masculine threat on negative affect is mediated by resilience. *p< 0.05, **p< 0.01, ***p< 0.001.

There was a significant indirect effect of masculine identity threat on negative affect through resilience, (ab_cs_ = .15, BCa CI [.07, .24], see Fig 4). The Sobel test confirmed there was significant mediation (Z = 3.97, *p* < .001). The mediator could account for roughly 35% of the total effect (*P*_m_ = .35).

## Discussion

This study investigated the association of perceived stress, cancer-related masculine identity threat, and resilience with quality of life and with positive and negative emotional adjustment in 204 men with localised prostate cancer. It also examined the moderating and mediating role of resilience in some of these pertinent relationships. Age, entered first in the analysis, explained a small proportion of variance on negative affect with younger men reporting greater negativity. Likewise, in other studies younger age was predictive of poorer psychological functioning [13, 48, 49]. This may reflect greater disruptive effects posed by the illness on careers, financial capacity, family life, and relationship intimacy in younger age groups. These men in particular should be counselled about potential post-treatment declines in emotional well-being. While length of time since diagnosis was not a significant predictor of any outcome, type of treatment did have an impact. Surgery predicted higher quality of life, and surgery with adjuvant treatment predicted lower quality of life. This might be expected given that adjuvant therapy extends the treatment period with potential added burden. While the variance explained was moderate, this concurs with studies of prostate cancer survivors showing that health-related quality of life varied by treatment with better scores for those having radical prostatectomy than external beam radiation [50], and lower physical quality of life in those who received external radiation therapy [13]. However, others found no such differences in quality of life [51, 52], and a systematic review [53] comparing those who received surgery and radiotherapy concluded that data on symptomatic and quality of life outcomes are of insufficient quality to offer clear guidance to men about risks to these aspects of their lives.

Results for psychological predictors reveal that for men diagnosed within the previous five years, perceived stress was the strongest predictor of their adjustment, it being related to lower quality of life and positive affect and to higher distress and negative affect. This accords with findings in men with newly diagnosed disease [25] and up to two years [26–28], and attests to the important role of stress in understanding both functional and emotional adjustment in survivorship over the longer term. Our previous research also demonstrated that perceived stress was the strongest and most consistent predictor of mood in men awaiting prostate biopsy [54] indicating its value both pre-diagnosis and across later phases of the prostate cancer experience. Similarly in studies of women with breast cancer, stress appraisal was a strong predictor of emotional adjustment [55–57] indicating its key contribution to emotional response to cancer for both men and women, and highlighting that stress appraisal is an important target for interventions. This strong impact of perceived stress suggests that screening for high levels at diagnosis is advisable to allow for timely psychological intervention. If neglected, high stress may continue to have a negative impact on quality of life and emotional adjustment up to five years later. Furthermore, an association reported between high perceived stress and prostate cancer-specific mortality [58] emphasises its value in cancer care.

While both cancer-related masculine threat and psychological resilience showed strong bivariate associations in the expected direction with all adjustment indices, the perceived stress variable outweighed their impact in multivariate analyses. Resilience and masculine threat predicted worse quality of life and emotional adjustment, but explained only a small amount of the variance in outcomes. Resilience and masculine identity threat did, however, explain additional amounts of variance on positive affect. Understanding predictors of positive emotions in cancer patients is important as promoting positive psychosocial outcomes is increasingly viewed to be just as critical as minimizing negative ones [59]. The relationships between resilience and distress and between masculine identity threat, distress and quality of life concur with previously reported findings [31, 38, 39]. In this study a robust measure of cancer-related masculine threat was used and we extended our understanding of the role of this threat on negative and positive affect.

The current study is the first to investigate the interaction of resilience with perceived stress and emotional status in patients with prostate cancer. It found that resilience moderated the positive relationship between stress and distress with those low in resilience being most vulnerable to the negative impact of perceived stress on distress. Resilience is viewed as an individual’s ability to maintain or restore relatively stable functioning when confronted with stressful life events, and this study confirms the suggestion by Sharpley et al. [31] that it acts as a buffer against distress in prostate cancer. While resilience has been increased by training in a small sample of women with breast cancer [60] the evidence is limited as very few resilience interventions have been developed and evaluated in cancer groups [61]. A resilience measure, however, could still be used as a screening tool to identify those who could benefit from stress management to alleviate distress.

Results here are the first to show that resilience mediates the relationship between cancer-related masculine threat and distress, and between masculine threat and negative affect. So after resilience is controlled, the effects of masculine identity threat on adjustment are diminished. Given the difficult functional side effects of treatments for prostate cancer, men can report a diminished sense of manhood [62] where masculinity may be rated against a hegemonic gender norm, characterised by expectations to be independent, self-reliant, in control, and strong [63]. While this gender role threat has been linked to poor outcomes at diagnosis [39] and two years on [38], more quantitative research is warranted to understand when and how this influences adjustment. This quantitative study extends the time-frame up to five years post diagnosis and suggests that resilience is a protective attribute that may help patients with prostate cancer to reinterpret perceived threats to their masculinity in such a way that it is not linked to poorer emotional adjustment.

### Limitations

There are a number of limitations to this study, it is cross-sectional in nature and so identification of causal relationships among variables is not possible. Prospective studies are thus needed. In addition, as the sample was self-selected (online survey) and data were self-reports, no reliable information could be obtained on medical status (e.g., Gleeson score). Over three quarters of the sample had third level education which impacts on generalisability of findings. Socioeconomic status which may influence adjustment was not measured. While measurement of quality of life was general and cancer-specific, the emotional adjustment measures were generic and there may have been recent events that affected the respondents emotions regardless of their illness. Notwithstanding these limitations, the results here provide useful insights into the current psychological status of men in survivorship and into potentially important predictors of quality of life and of positive and negative emotional adjustment in the five years following diagnosis.

### Conclusions

In conclusion findings show that perceived stress was the most powerful predictor of adjustment in the model thus adding to the literature supporting the importance of stress appraisal as a predictor of adaptation in men with prostate cancer. Furthermore, psychological resilience was shown to be both an explanatory and a protective factor. This supports the clinical importance of resilience, in tandem with threat and stress, in predicting risk of emotional distress among prostate cancer patients up to five years post diagnosis. Referral for timely psychological intervention will lead to more effective patient management and care.
